# Reduced Warburg Effect in Cancer Cells Undergoing Autophagy: Steady- State ^1^H-MRS and Real-Time Hyperpolarized ^13^C-MRS Studies

**DOI:** 10.1371/journal.pone.0092645

**Published:** 2014-03-25

**Authors:** Gigin Lin, Gabriela Andrejeva, Anne-Christine Wong Te Fong, Deborah K. Hill, Matthew R. Orton, Harry G. Parkes, Dow-Mu Koh, Simon P. Robinson, Martin O. Leach, Thomas R. Eykyn, Yuen-Li Chung

**Affiliations:** 1 Cancer Research UK and EPSRC Cancer Imaging Centre, Division of Radiotherapy and Imaging, The Institute of Cancer Research and Royal Marsden Hospital, Sutton, Surrey, United Kingdom; 2 Department of Medical Imaging and Intervention, Chang Gung Memorial Hospital at Linkou, College of Medicine, Chang Gung University, Taoyuan, Taiwan; 3 Division of Imaging Sciences and Biomedical Engineering, King′s College London, The Rayne Institute, St Thomas Hospital, London, United Kingdom; Texas Tech University Health Sciences Center, United States of America

## Abstract

Autophagy is a highly regulated, energy dependent cellular process where proteins, organelles and cytoplasm are sequestered in autophagosomes and digested to sustain cellular homeostasis. We hypothesized that during autophagy induced in cancer cells by i) starvation through serum and amino acid deprivation or ii) treatment with PI-103, a class I PI3K/mTOR inhibitor, glycolytic metabolism would be affected, reducing flux to lactate, and that this effect may be reversible. We probed metabolism during autophagy in colorectal HT29 and HCT116 Bax knock-out cells using hyperpolarized ^13^C-magnetic resonance spectroscopy (MRS) and steady-state ^1^H-MRS. 24 hr PI103-treatment or starvation caused significant reduction in the apparent forward rate constant (k_PL_) for pyruvate to lactate exchange compared with controls in HT29 (100 μM PI-103: 82%, p = 0.05) and HCT116 Bax-ko cells (10 μM PI-103: 53%, p = 0.05; 20 μM PI-103: 42%, p<0.0001; starvation: 52%, p<0.001), associated with reduced lactate excretion and intracellular lactate in all cases, and unchanged lactate dehydrogenase (LDH) activity and increased NAD+/NADH ratio following PI103 treatment or decreased LDH activity and unchanged NAD+/NADH ratio following starvation. After 48 hr recovery from PI103 treatment, k_PL_ remained below control levels in HT29 cells (74%, p = 0.02), and increased above treated values, but remained below 24 hr vehicle-treated control levels in HCT116 Bax-ko cells (65%, p = 0.004) both were accompanied by sustained reduction in lactate excretion, recovery of NAD+/NADH ratio and intracellular lactate. Following recovery from starvation, k_PL_ was significantly higher than 24 hr vehicle-treated controls (140%, p = 0.05), associated with increased LDH activity and total cellular NAD(H). Changes in k_PL_ and cellular and excreted lactate provided measureable indicators of the major metabolic processes accompanying starvation- and drug-induced autophagy. The changes are reversible, returning towards and exceeding control values on cellular recovery, which potentially identifies resistance. k_PL_ (hyperpolarized ^13^C-MRS) and lactate (^1^H-MRS) provide useful biomarkers for the autophagic process, enabling non-invasive monitoring of the Warburg effect.

## Introduction

Autophagy is a lysosome-dependent reversible catabolic cellular response activated in starvation or stress whereby proteins, organelles and cytoplasm are sequestered within double-membrane autophagosomes and subsequently digested and recycled to sustain cellular metabolism [1]. Autophagy is critical for maintaining cellular homeostasis and is a highly regulated process that can replenish depleted energy stores during starvation by removal and degradation of cytoplasmic components. However, prolonged activation of autophagic pathways can lead to the depletion of organelles and critical proteins which may result in cell death [2], [3]. Autophagy has been investigated in many research fields, including cancer [2]–[4], cardiovascular disease [5] and neurodegeneration [6], since on the one hand it provides a biological protection mechanism in response to cellular stresses but on the other it can also contribute to cell death mechanisms. This process could paradoxically allow cancer cells to survive in hostile environments and aid recovery once the stress is removed, providing a potential mechanism of resistance to therapy [4]. Some anti-cancer therapies, such as PI3K/mTOR inhibitors, are known to induce autophagy in cancer cells [7] and may also induce autophagy in tumors, potentially prolonging tumor survival [4], [8]. Currently, autophagy is best assessed by observation of double-membrane autophagic vacuoles by electron microscopy (EM) and western blotting of the conversion of ubiquitin-like protein LC3I to LC3II [9]. There are currently no non-invasive methods to monitor induction of autophagy or subsequent recovery from autophagy. Furthermore, the metabolic changes accompanying autophagy and recovery from this process are poorly understood.

Cancer cells often exhibit enhanced aerobic glycolysis, also known as the Warburg effect, with increased transcriptional regulation of a number of glycolytic enzymes including lactate dehydrogenase-A (LDH-A). Increased Warburg effect has been shown to drive both tumor growth and the spread of metastases and is associated with poor outcome in cancer [10]. Autophagy involves many major metabolic processes, some of which are regulated by oncogenic signaling pathways. There is considerable interplay between autophagic control points and key nodes in oncogenic signaling pathways, leading to pathway inhibitors in some cases directly affecting the autophagic process, or indirectly modulating the same metabolic pathways that are induced by autophagy [11], [12]. For example, the inhibition of mTORC1 is a key driver of the induction of autophagy in cancer cells [11], [12]. Cellular stress arising from shortage of amino acids or direct PI3K inhibition could cause autophagy via inhibition of mTORC1 with both of these processes causing metabolic effects in addition to those arising directly from autophagy. These situations could also be encountered during cancer treatment in patients.

Magnetic Resonance Imaging (MRI) is widely used for imaging in medicine and MR Spectroscopy (MRS) provides chemically specific analysis of metabolite concentrations in cell extracts, whole cells, tissue biopsies and *in vivo* [13]. Recent advances in hyperpolarized ^13^C-magnetic resonance spectroscopy (MRS) employing Dynamic Nuclear Polarization (DNP) have enabled significant enhancement of the intrinsic ^13^C-MRS signal by many orders of magnitude in a number of metabolites and has enabled real-time measurements of the kinetics of a range of important endogenous enzyme reactions both *in vitro* in suspensions of viable whole cells and *in vivo* in tumors [14]. The apparent exchange rate constant of hyperpolarized [1-^13^C] pyruvate to lactate (k_PL_) provides a potential metabolic biomarker for diagnosis [15] and for assessing treatment response [16]–[20]. k_PL_ has also been shown to decrease following drug induced cell death, attributed to apoptosis with the activation of poly(ADP-ribose) polymerase (PARP) and depletion of the cofactors nicotinamide adenine dinucleotide (NAD(H)) [14].

The TCA cycle is expected to become more active during autophagy, as the amino acids and fatty acids generated by the autophagic process are utilized to sustain energy homeostasis [21], leading to modulation of aerobic glycolysis in autophagic cells. We hypothesized that these changes would lead to a reduction in flux from pyruvate to lactate, which could be reversed if cells recovered from autophagy. In this study, we measured the apparent [1-^13^C] pyruvate to lactate exchange rate, k_PL_, by DNP and ^13^C-MRS, in order to develop a non-invasive biomarker of drug-induced autophagy and subsequent recovery from autophagy, and to gain further insights into the metabolic changes accompanying drug-induced autophagy. We investigated the longitudinal metabolic changes associated with drug-induced autophagy and its recovery and compared them with the metabolic changes associated with the established model of autophagy, induction by starvation. We used two cell lines, HT29 and Bax-deficient HCT116 colon carcinoma (HCT116 Bax-ko) [22], in these studies. HCT116 Bax-ko cells have impaired apoptosis pathways allowing evaluation of the metabolic changes associated with drug-induced autophagy in the absence of apoptosis. Autophagy was induced in these cells either by serum and amino acid starvation with Hanks' balanced salt solution (HBSS) media, or by treatment with PI-103, a class I PI3K/mTOR inhibitor [7].

Our study confirmed our hypotheses, showing that cells undergoing autophagy had reduced k_PL_ measured with DNP and ^13^C-MRS, together with reduced intracellular lactate and lactate excretion measured by ^1^H-MRS, reflecting a reduced Warburg effect during starvation- and drug-induced autophagic cellular processes. These important metabolic effects are reversed when cells recover from starvation- and drug-induced autophagy, providing a potential means of identifying resistance. The results show that the measurements of k_PL_ by hyperpolarized ^13^C-MRS, as well as lactate by ^1^H-MRS provide sensitive biomarkers of the metabolic effects associated with starvation- and drug-induced autophagy together with its recovery, providing critical information on an important aspect of cancer cell metabolism.

## Materials and Methods

### Cell culture

All media and reagents for cell culture were purchased from Life Technologies. HT29 cells (from American Type Culture Collection, ATCC) were cultivated in McCoy 5A medium with glutamine and HEPES. HCT116 Bax-ko cells (a kind gift from Dr. Bert Vogelstein, Johns Hopkins Medical Center, USA; via Dr. Paul Clarke, ICR, Sutton, UK [23]) were cultivated in Dulbecco's Modified Eagle Medium with glutamine, and non-essential amino acids. 10% heat inactivated FCS with 100 U/mL penicillin and 100 μg/mL streptomycin were added in all medium. Cells were maintained at 37°C in a humidified 5% CO_2_ atmosphere and growth media replenished every 48 hours. HT29 cells were treated with 100 μM PI-103 for 24 hr. HCT116 Bax-ko cells were treated with HBSS medium for 6 or 24 hr and with 10 μM or 20 μM PI-103 for 24 hr.

For the cellular and metabolic analysis of cells recovered from autophagy, 24 hr HBSS or 20 μM PI-103-treated HCT116 Bax-ko cells and 100 μM PI-103-treated HT29 cells were maintained for a further 48 hr in normal DMEM (with glutamine, non-essential amino acids, 10% fetal calf serum and penicillin added) or McCoy 5A medium (with glutamine and HEPES) culture media under standard conditions, respectively.

### Cell-cycle analysis

2×10^6^ cells were fixed with 70% ethanol for 30 min at 4°C, incubated with 100 μg/ml RNAase and 40 μg/ml PI in phosphate-buffered saline for 301min at 37°C. DNA histograms were generated by FACS analysis.

### Annexin V/PI analysis

5×10^5^ cells were resuspended in binding buffer, incubated at 25°C for 5 min in the dark with 5 μL fluorescein isothiocyanate (FITC)–annexin-V and 5 μL PI (BioVision), and analyzed by Fluorescence Activated Cell Sorting (FACS, BD LSRII flow cytometer) to determine annexin binding and PI exclusion.

### Western blotting

Cell lysates were analyzed by western blotting as described previously [22]. Cell lysate protein was transferred onto Immobilon-P membranes (Millipore; Bedford MA, USA). Blots were blocked in 5% non-fat milk or 5% bovine albumin and then incubated with primary antibody for pS6RP (Cell Signaling), S6RP (Cell Signaling), p4E-BP1 (Cell Signaling), total 4E-BP1 (Cell Signaling), pAkt (Cell Signaling), total Akt (Cell Signaling), cleaved PARP (Cell Signaling), caspase 3 (Cell Signaling), LC3 (Cell Signaling), MCT-1 (Millipore) or MCT-4 (Santa Cruz Biotechnology). The membranes were then incubated with anti-rabbit secondary antibody (GE Healthcare). Western blots for α-tubulin (Cell Signaling) provided a loading control. Specific binding antibody-target protein interactions were detected using enhanced chemiluminescence plus reagents (Amersham Biosciences, Buckingham-shire, UK) and exposure to either Hyperfilm ECL (Amersham) or XOMAT Kodak (Rochester NY, USA) autoradiography film.

### Lactate dehydrogenase enzymatic assay

Total cellular LDH activities were measured using standard spectro-photometric methods [24]. Cells were collected by standard trysin treatment, lysed on ice in extraction buffer (Triethanolamine/HCL 50 mM, EDTA 1 mM, MgCl_2_ 2 mM, mercaptoethanol 26 mM) and centrifuged at 16000 g for 10 min to give total protein concentrations in the supernatants of about 5 mg/ml. Reactions were started by adding assay solutions (Triethanolamine/HCL 60 mM, NADH 0.17 mM, Sodium pyruvate 0.4 mM, Triton 0.05%), and the activities of LDH enzymes were measured spectrophotometrically at 340 nm, with enzymatic activities measured as nmol/min per million of cells.

### NAD+/NADH measurement

Total cellular NAD+/NADH ratios were measured using an NAD+/NADH quantification kit (BioVision, Mountain View, CA, USA), according to the protocol provided.

### Electron microscopy

Cells were grown in 13 mm dishes and fixed for 4 hours in 2.5% glutaraldehyde fixative in phosphate buffer at 4°C. After fixation, cell monolayers were placed in glutaraldehyde wash solution and then post-fixed in Millonigs Osmium Tetroxide fixative for 15–30 minutes. The cultures were dehydrated through a graded series of 10% to 100% ethanol, infiltrated, and then embedded in medium TAAB Premix Resin. Ultrathin sections were collected on copper grids and stained with uranyl acetate and lead citrate. Sections were viewed using a Hitachi H7600 transmission electron microscope.

### DNP ^13^C-MRS studies

[1-^13^C]pyruvic acid containing 15 mM OX63 free radical was polarized for 1 hr in a HyperSense DNP polarizer (Oxford Instruments Molecular Biotools Ltd, Abingdon, UK) at 3.35T and 1.4 K as described previously [25]. The polarized sample was dissolved in a phosphate buffered solution containing 50 mM unlabeled lactate, EDTA and NaOH to achieve a final pH of 7. 100 μL of this mixture was added to a 500 μL suspension of cells (∼40–80 million cells) in a 5 mm NMR tube. The final concentration of polarized pyruvate was 8 mM, after which serial ^13^C-MRS spectra were acquired in a BBO probe every 2 sec with a 10° radio-frequency pulse on a 500 MHz Bruker NMR system (Bruker Biospin, Coventry, UK). Cells were maintained at 37°C throughout. Serial spectra were phase and baseline corrected, and integrated using Topspin software (Bruker Biospin, Coventry, UK). The integrated peak integrals in the pyruvate/lactate experiments were plotted as a function of time and least squares-fitting was carried out in Matlab (The Mathworks Inc, Natick, MA, USA). The apparent rate constants were obtained by simultaneously fitting pyruvate and lactate integrals to the modified Bloch equations using a two-site exchange model incorporating flip-angle correction as previously described [25]. The apparent rate constants (k_PL_) for the forward reaction of conversion of pyruvate to lactate are normalized to cell number.

### ^1^H-MRS °f culture medium and cell extracts

Cells were extracted by dual phase extraction procedures as previously described [26]. Water-soluble extracts were freeze-dried and reconstituted in 700 μL deuterated water (D_2_O, Sigma Aldrich) and the extracts (500 μl) placed in 5 mm NMR tubes. 50 μL of 0.75% sodium 3-trimethylsilyl-2,2,3,3-tetradeuteropropionate (TSP) in D_2_O (Sigma Aldrich) was added to the samples for chemical shift calibration and quantification. Culture media samples from cells following various treatment regimes were also analyzed by ^1^H-MRS, collected before cells were harvested for cellular extractions. 500 μL of media sample and 50 μL of D_2_O were placed in the NMR tube with 50 μL of 0.75% TSP in D_2_O for chemical shift calibration and quantification. Spectral assignments were based on literature values [27]. ^1^H spectra were acquired with a Bruker 500 MHz spectrometer (Germany) with 7500 Hz spectral width, 16384 time domain points, 128 scans, temperature 298K, acquisition time approximately 5 minutes. The water resonance was suppressed by gated irradiation centered on the water frequency. Spectral processing was carried out using the Topspin-2 software package (Bruker Biospin, Coventry, UK), and metabolite concentrations were quantified and normalized to cell number.

### Statistical analysis

Data are presented as the mean ± standard error of mean. For comparison of metabolic flux, metabolite concentrations and ratios, the unpaired Student's t-test was used with a P value of ≤0.05 considered to be statistically significant. All statistical tests were two-sided.

## Results

### PI-103-treatment and starvation causes reversible autophagy in HT29 and HCT116 Bax-ko cells

**Figure 1:**
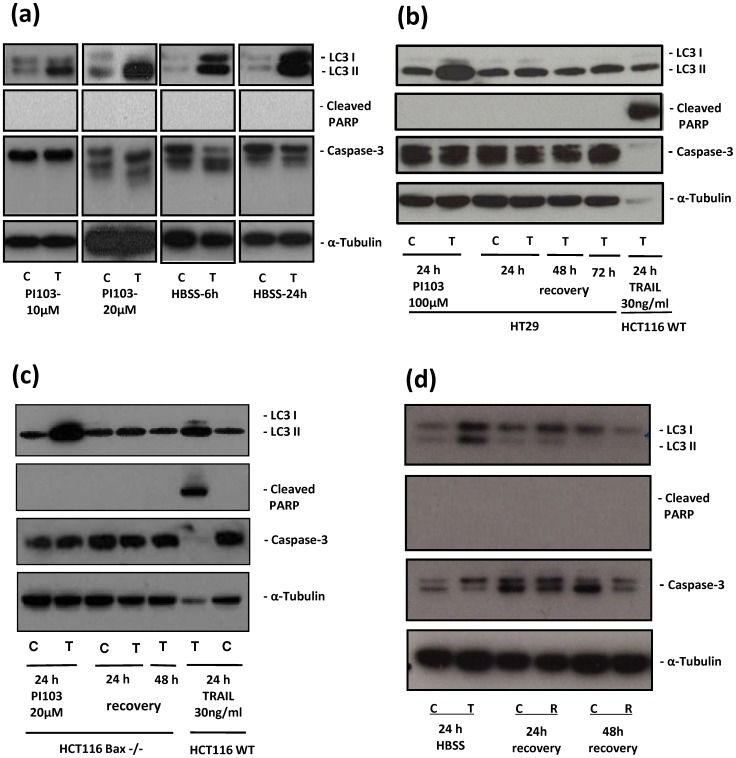
Autophagy is induced in HT29 and HCT116 Bax-ko cells and is reversed after treatment ends. (a) Western blots of LC3, Cleaved PARP, Caspase 3 and α-tubulin in control HCT116 Bax-ko cells and in 10 μM or 20 μM PI-103-treated or 6 hr and 24 hr HBSS-treated HCT116 Bax-ko cells. (b) Western blots of LC3, Cleaved PARP, Caspase 3 and α-tubulin in control HT29 cells and in 24 hr 100 μM PI-103-treated HT29 cells and at 24 hr, 48 hr and 72 hr of their recovery. TRAIL-treated HCT116 WT cells are used as positive control for apoptosis, as TRAIL has been reported to induce apoptosis in HCT116 WT cells [22]. As expected, the presence of cleaved PARP and highly reduced caspase 3 expressions were observed in 24 hr TRAIL-treated HCT116 WT cells when compared with vehicle-treated controls. (c) Western blots of LC3, Cleaved PARP, Caspase 3 and α-tubulin in control HCT116 Bax-ko cells and in 24 hr 20 μM PI-103-treated HCT116 Bax-ko cells and at 24 hr and 48 hr of their recovery. TRAIL-treated HCT116 WT cells are used as positive control for apoptosis. (d) Western blots of LC3, Cleaved PARP, Caspase 3 and α-tubulin in control HCT116 Bax-ko cells and in 24 hr of HBSS-treated HCT116 Bax-ko cells and at 24 and 48 hr of their recovery.

Induction of autophagy was observed in HCT116 Bax-ko and HT29 cells treated with PI-103, as confirmed by the over-expression of LC3II in western blots (Figure 1a and b). Induction of autophagy and over-expression of LC3II was also found in HCT116 Bax-ko cells after 6 and 24 hr of starvation in HCT116 Bax-ko cells (Figure 1a). A higher level of LC3I and LC3II expression was seen after 24 hr of starvation when compared with 6 hr starvation. Following removal of PI-103 treatment or replenishment of normal growth medium following 24 hr of PI-103 treatment or starvation, respectively, the over-expression of LC3II returned to basal levels after 48 hr of recovery (Figure 1b-d). Hence, the 48 hr time-point following 24 hr starvation or PI-103 treatment was chosen for the DNP and ^1^H-MRS recovery measurements. There was no indication of apoptosis as shown by the lack of cleaved PARP or change in caspase 3 expressions in PI-103-treated or starved cells (Figure 1a-d). In order to obtain a positive control for apoptosis, apoptosis was induced in HCT116 wild type (WT) cells by TRAIL treatment, as TRAIL has been reported to induce apoptosis in HCT116 WT cells [22]. As expected, the presence of cleaved PARP and reduced caspase 3 level were observed in TRAIL-treated HCT116 WT cells when compared with vehicle-treated controls (Figure 1b and c).

**Figure 2:**
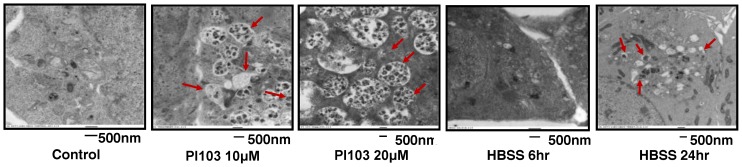
Electron microscopy showing autophagic vacuoles. Electron microscopy images of autophagic vacuoles in control, 10 μM or 20 μM PI-103-treated or 6 hr and 24 hr HBSS-treated HCT116 Bax-ko cells. Red arrows illustrate some of the autophagic vacuoles at different stages of the autophagy process.

The presence of autophagic vacuoles was confirmed by electron microscopy performed in starved (as double-membrane autophagosomes) or PI-103-treated (predominantly as later stage autolysosomal structures) HCT116 Bax-ko cells, indicating the induction of autophagy (Figure 2). Autophagic vacuoles were absent in control cells.

**Figure 3:**
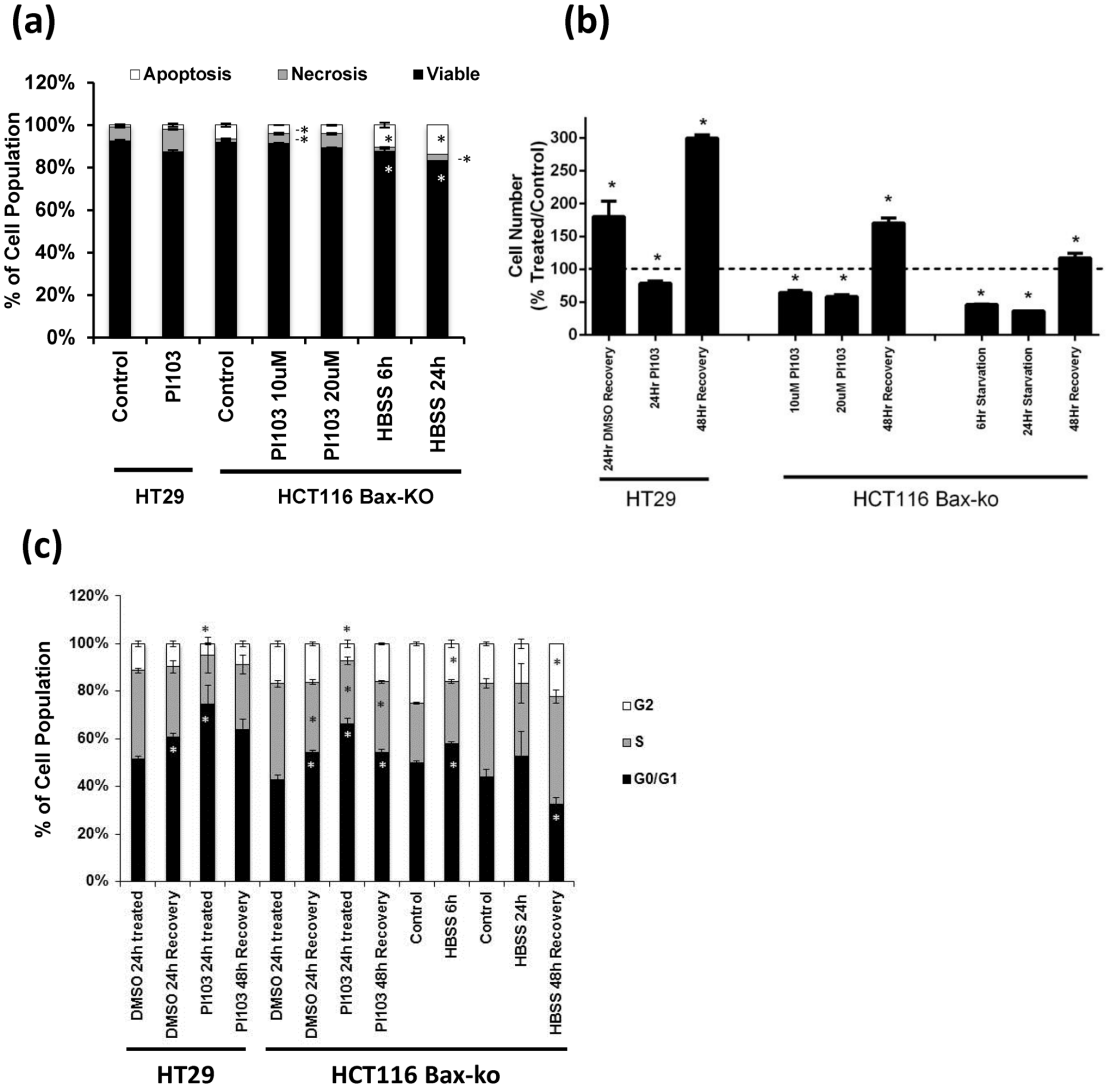
Cellular characterization of starvation- and PI-103-induced autophagic cells. (a) Annexin V and propidium iodide staining (PI) assay measuring the proportion of cells undergoing apoptosis and necrosis in HT29 cells following 24 hr of 100 μM PI-103 treatment and HCT116 Bax-ko cells following 24 hr of 10 μM or 20 μM PI-103 treatment, or 6 hr or 24 hr of starvation. Data are expressed as means of percentage ± s.e.m. (minimum *n* = 3 in each group). Statistically significant changes are indicated (*p<0.05). (b) Cell number compared with 24 hr DMSO-treated controls in HT29 cells following 24 hr DMSO-treated recovery, 24 hr of 100 μM PI-103 treatment and its 48 hr recovery and 24 hr vehicle-treated controls in HCT116 Bax-ko cells following 24 hr of 10 μM or 20 μM PI-103 treatment, or 6 hr or 24 hr of starvation and its recovery from 24 hr of 20 μM PI-103 treatment or starvation. Data are expressed as means of percentage ± s.e.m. (minimum *n* = 3 in each group). Statistically significant changes are indicated (*p<0.05). (c) Cell cycle analysis. Data are means of percentage ± s.e.m. HT29 cells 24 hr DMSO-treated control, 24 hr DMSO-recovered control, 24 hr PI-103-treated 100 μM, 24 hr PI-103-recovered, 48 hr PI-103-recovered, (*n* = 3 in each group); HCT116 Bax-ko cells 24 hr DMSO-treated control, 24 hr DMSO-recovered control, 24 hr PI-103-treated 20 μM, 24 hr PI-103-recovered, 48 hr PI-103-recovered, (*n* = 3 in each group); HCT116 Bax-ko cells 6 hr full media control, 6 hr starvation (HBSS), 24 hr full media control, 24 hr starvation (HBSS), 48 hr HBSS-recovered (*n* = 3 in each group). *P<0.01.

Levels of apoptosis and necrosis in HT29 and HCT116 Bax-ko cells under starvation and PI-103 treatment were assessed in adherent cells by annexin V/PI analysis (Figure 3a) demonstrating a majority of viable cells with a small percentage of necrotic (HT29 DMSO-treated (vehicle-control): 6.5±2.2%; 100 μM PI-103: 10.7±2.7% (p = 0.29); HCT116 Bax-ko DMSO-treated (vehicle-control): 1.5±0.1%; 6 hr starved: 1.9±0.2% (p = 0.06) and 24 hr starved: 3.0±0.4% (p = 0.01); 10 μM PI-103: 4.6±0.4% (p = 0.02) and 20 μM PI-103: 6.6±0.4% (p = 0.79)) or apoptotic cells (HT29 DMSO-treated: 1.0±0.7%; 100 μM PI-103: 2.0±1.4% (p = 0.54); HCT116 Bax-ko vehicle-control: 6.6±0.5%; 6 hr starved: 10.5±0.8% (p = 0.001) and 24 hr starved: 13.8±0.8% (p = 0.003); 10 μM PI-103: 4.0±0.1% (p = 0.26) and 20 μM PI-103: 4.1±0.2% (p = 0.30)) in control and treated groups. No floating cells were observed in any of the treated groups.

A reduced number of adherent cells was found in all treatment groups following 24 hr of 100 μM treatment in HT29 cells (78±2%, p<0.01), 6 hr and 24 hr of starvation (46±0.1% and 36±0.4%, respectively, p<0.01) or 24 hr of 10 μM and 20 μM PI-103 treatment (65±0.1% and 58±0.1%, respectively, p<0.01) in HCT116 Bax-ko cells when compared with controls (Figure 3b). An increase in the number of adherent cells above the 24 hr vehicle-treated control levels was observed in PI-103-induced (170±8%, p<0.05 (HCT116 Bax-ko); 303±16%, p<0.01 (HT29)) and starvation-induced (117±7%, p<0.05) and autophagic cells following 48 hr of recovery (Figure 3b), indicating that the cell population increased above control levels following recovery from autophagy. The 24 hr vehicle treated cells were used as controls for both the treatment and recovery groups, as the control cells would be too confluent by 48 hr of recovery.

Cell cycle analysis by flow cytometry was performed on each treatment group, and after 24 hr and 48 hr of recovery (Figure 3c). A 24 hr recovery time-point for DMSO-treated controls was also included for each PI-103 experiment as the cell numbers of these groups are similar to the cell numbers of PI-103-treated cells following 48 hr of recovery. G1 arrest was observed in HT29 and HCT116 Bax-ko cells after treatment with PI-103, returning to the 24 hr DMSO-treated level by 48 hr of recovery (Figure 3c). Increased G1 phase was also found in 24 hr recovered DMSO-treated HT29 (9±3%, p = 0.01) and HCT116 Bax-ko (11±1%, p = 0.004) cells when compared with 24 hr DMSO-treated cells. The percentages of 24 hr recovered DMSO-treated cells at each phase of the cell cycle are very similar to the 48 hr recovered PI-103-treated time-point in both HT29 and HCT116 Bax-ko cells (Figure 3c). This may be due to the recovered control (DMSO-treated) and PI-103-treated cells being more confluent at these time-points, which is consistent with their comparable cell numbers.

No change in the cell cycle profile of HCT116 Bax-ko cells was observed following 6 or 24 hours of starvation (Figure 3c). A decrease in G1 (12±4%, p = 0.04) and an increase in G2 phase (5±1%, p = 0.003) were found in 48 hr recovered HCT116 Bax-ko cells when compared with 24 hr media-treated control cells and the cell numbers are similar between these two groups (Figure 3c). No significant change in cell size was observed in any of the treatment groups when compared with controls.

### PI-103 treatment reduces AKT and mTOR pathway activation in HT29 and HCT116 Bax-ko cells and this process is reversible

**Figure 4:**
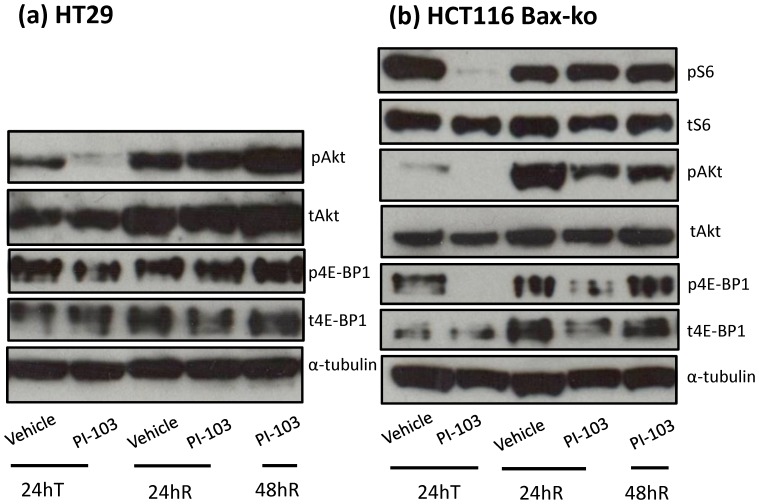
The AKT/mTOR pathway is down-regulated in PI-103-treated HT29 (a) and PI-103 (b) cells and recovered by 48 hr of recovery from PI-103 treatment. Western blots of pS6 ribosomal protein, total S6 ribsomal protein, p4E-BP1, total 4E-BP1, pAkt, total Akt and α-Tubulin (loading control) in PI-103-treated and its recovery in HT29 and HCT116 Bax-ko cells.

The effects of PI-103 treatment on the AKT and mTORC pathways were examined in HT29 and HCT116 Bax-ko cells during treatment and during 24 and 48 hr of recovery. Decreased expression of pAkt and p4E-BP1 was seen in HT29 and HCT116 Bax-ko cells following 24 hr of PI-103 treatment with expression of these proteins returning to pre-treatment levels after 48 hr of recovery (Figure 4a and b). Reduced pS6 expression was also found in PI-103-treated HCT116 Bax-ko cells and its level recovered from 24 hr of recovery (Figure 4b). Taken together, these data indicate that treatment with PI-103 inhibits the AKT and mTOR pathway in both cell lines and this process is reversible when the drug is removed and the cells are allowed to recover.

### Real-time apparent ^13^C-pyruvate to lactate exchange rates are reduced in PI-103-and starvation-induced autophagy, remaining reduced following recovery from PI-103 but increasing with recovery from starvation

**Figure 5:**
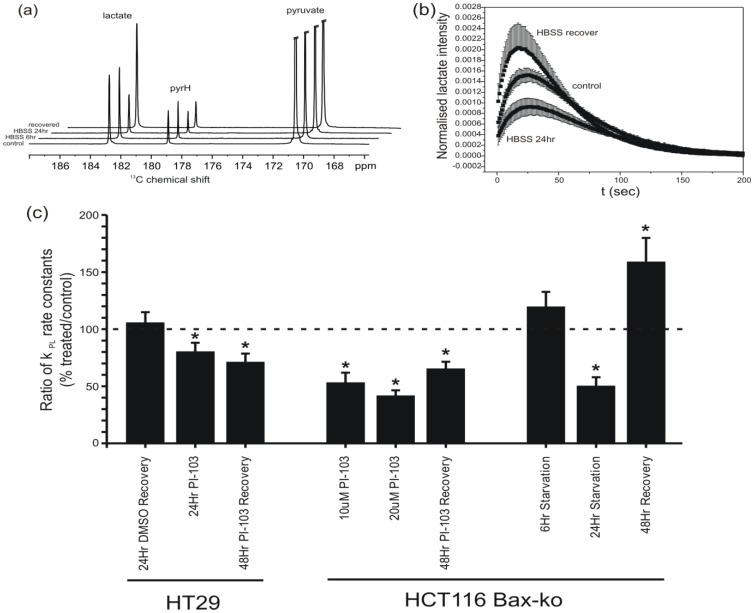
Assessment of apparent ^13^C-pyruvate to lactate exchange rate. (a) Hyperpolarized ^13^C-spectrum from an HCT116 Bax-ko cell assay showing the sum over the entire dynamic time series from a control cell experiment, following 6 hr starvation, 24 hr starvation with HBSS media and after 24 hr starvation followed by 48 hr cell recovery. The spectrum displays peaks from pyruvate (Pyr), lactate (Lac) and pyruvate hydrate (Pyr H). (b) Plot of the lactate peak integral as a function of time normalized to the pyruvate peak integral at time t = 0 s and normalized per million cells acquired for HCT116 Bax-ko control cells, 24 hr starvation with HBSS media and 24 hr starvation followed by 48 hr recovery. (c) The mean ratios (treated/24 hr vehicle-control) of the apparent forward reaction rate of pyruvate to lactate exchange k_PL_ (derived from fitting of the experimental data and normalized to cell number) (± s.e.m.) for 24 hr DMSO-treated recovery, 100 μM PI-103 treated and 48 hr recovery following 100 μM PI-103 treatment in HT29 cells; for 10 μM PI-103 treated, 20 μM PI-103 treated and 48 hr recovery following 20 μM PI-103 treatment in HCT116 Bax-ko cells; and for 6 hr HBSS starvation, 24 hr HBSS starvation and 24 hr starvation followed by 48 hr recovery in HCT116 Bax-ko cells. Minimum *n* = 3 in each group. Statistically significant changes are indicated (*p≤0.05).

Hyperpolarized [1-^13^C]pyruvate to lactate exchange was monitored in real-time by ^13^C-MRS to measure exchange kinetics in autophagic cells. Figure 5a shows average ^13^C-MR spectra calculated for each group by summing over the entire dynamic time-series for HCT116 Bax-ko cell suspensions after the addition of hyperpolarized [1-^13^C]pyruvate in control, 24 hr starvation and 48 hr of recovery from autophagy. Figure 5b shows the corresponding time-series of the normalized peak integral of the lactate signal in the same groups. Apparent exchange rate constant k_PL_ were measured by non-linear least squares fitting to the dynamic data in each group and the data are presented as ratios (treated/24 hr vehicle control) in Figure 5c.

Decreased k_PL_ were observed in HT29 cells treated with PI-103 (82±7% of control; p = 0.05) (Figure 5c). No significant difference in k_PL_ was found between the 24 hr DMSO vehicle treated group and the 24 hr recovered DMSO-vehicle treated group (Figure 5c) in HT29 cells, despite the cell number and the population of cells in G1 phase being higher in the 24 hr recovered DMSO-treated group (Figure 3b and c). Hence, k_PL_ in the recovery groups was compared with its respective 24 hr vehicle-treated controls. k_PL_ remained lower in HT29 cells following 48 hr of recovery from PI-103 treatment (74±4% of control; p = 0.02), when compared with 24 hr DMSO-treated controls (Figure 5c).

Reduced k_PL_ were found in HCT116 Bax-ko cells treated with 10 μM (52.5±9.4% of control; p = 0.05) and 20 μM PI-103 (41.1±5.3% of control; p<0.0001) when compared with DMSO-vehicle controls (Figure 5c). Following 48 hr of recovery the apparent rate constant was elevated compared to treatment rates but did not return to 24 hr vehicle-treated control rates (65±7% of control; p = 0.004) (Figure 5c).

No significant change in k_PL_ was observed in HCT116 Bax-ko cell after 6 hr of starvation (121±15% of control; p = 0.21), whereas k_PL_ decreased significantly after 24 hr of starvation (52±5% of control; p<0.001) (Figure 5c). Following 48 hr of recovery after 24 hr of starvation, the apparent rate constants recovered to a level significantly (140% of control; p = 0.05) higher than 24 hr vehicle-treated controls (Figure 5c).

### PI-103- and starvation-induced autophagy results in reduced intracellular lactate and lactate excretion with a marked increase on recovery from starvation

**Figure 6:**
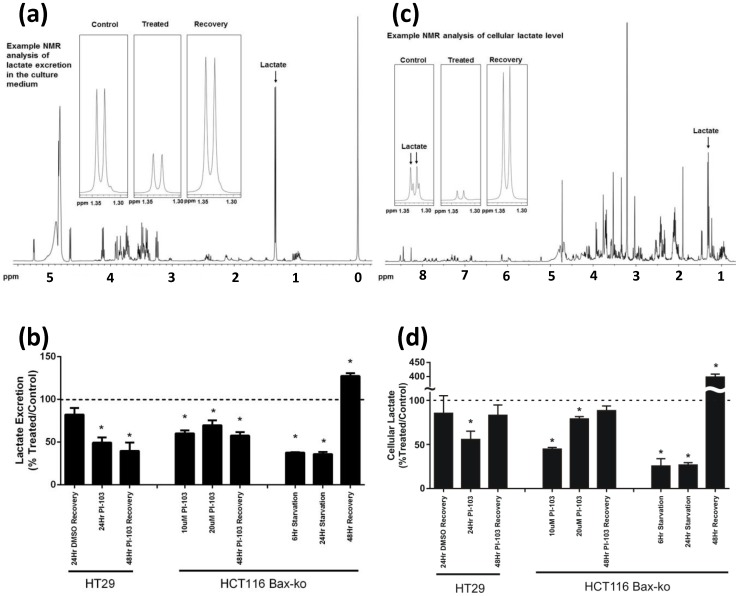
Levels of lactate excretion and intracellular lactate in PI-103- and HBSS-treated and its recovery in HT29 and HCT116 Bax-ko cells. Example 1H-MRS spectrum of a culture medium (a) and a cell extract (c) sample from HCT116 Bax-ko cells, with the expanded lactate signals are shown for control, 24 hr starved and 48 hr recovered HCT116 Bax-ko cells. Levels of (b) excreted lactate and (d) intracellular lactate in PI-103-treated or HBSS-treated HT29 and HCT116 Bax-ko cells and levels at 48 hours recovery from 24 hr of PI-103-treatment or starvation. Data are expressed as means of percentage ± s.e.m. Minimum *n* = 3 in each group. Statistically significant changes are indicated (*p≤0.05).

Total lactate excretion was measured in the culture media using ^1^H-MRS; an example spectrum is shown in Figure 6a. Reduced steady-state lactate excretion was found in HT29 cells treated with PI-103 (50±5% of control; p = 0.0007) and in HCT116 Bax-ko cells treated with PI-103 (10 μM: 60±3% of control; p = 0.002; 20 μM: 72±3% of control; p<0.0001) or following starvation (6 hr: 33±1% of control; p<0.0001; 24 hr: 36±2% of control; p<0.0001) (Figure 6b). No significant difference in the lactate excretion level was found between the 24 hr DMSO-treated group and the 24 hr recovered DMSO-treated group (Figure 6b) in HT29 cells, despite the cell number and the population of cells in the G1 phase being higher in the 24 hr recovered DMSO-treated (Figure 3b and c). Hence the lactate excretion level in the recovery groups was compared with its respective 24 hr vehicle-treated controls. Lactate excretion was lower than 24 hr DMSO-treated control levels after 48 hr recovery from PI-103 treatment (HT29: 40±10% of control; p = 0.002; HCT116 Bax-ko: 58±2% of control; p<0.0001) or was significantly increased from control (24 hr full media-treated) values after 48 hr recovery in the starvation group (127±3% of control; p = 0.002) (Figure 6b).

An example 1H-MRS spectrum of a cell extract is illustrated in Figure 6c. Reductions in intracellular lactate were also observed in PI-103-treated HT29 (55±3% of control; p = 0.04) and in HCT116 Bax-ko cells (10 μM: 45±2% of control; p<0.0001; 20 μM: 70±4% of control; p<0.0001) and HBSS-treated groups (6 hr: 26±8% of control; p = 0.001; 24 hr: 27±3% of control; p<0.0001) (Figure 6d). No significant difference in intracellular lactate was found between the 24 hr DMSO-treated group and the 24 hr recovered DMSO-treated group (Figure 6d) in HT29 cells. Hence, intracellular lactate level in the recovery groups was compared with its respective 24 hr vehicle-treated controls. Intracellular lactate returned to control levels in both HT29 and HCT116 Bax-ko cells after 48 hr recovery from PI-103-induced autophagy (Figure 6d). Intracellular lactate was found to be substantially increased in cells recovered from starvation-induced autophagy for 48 hr (398±10% of control; p<0.0001), compared with 24 hr vehicle-treated controls (Figure 6d).

**Figure 7:**
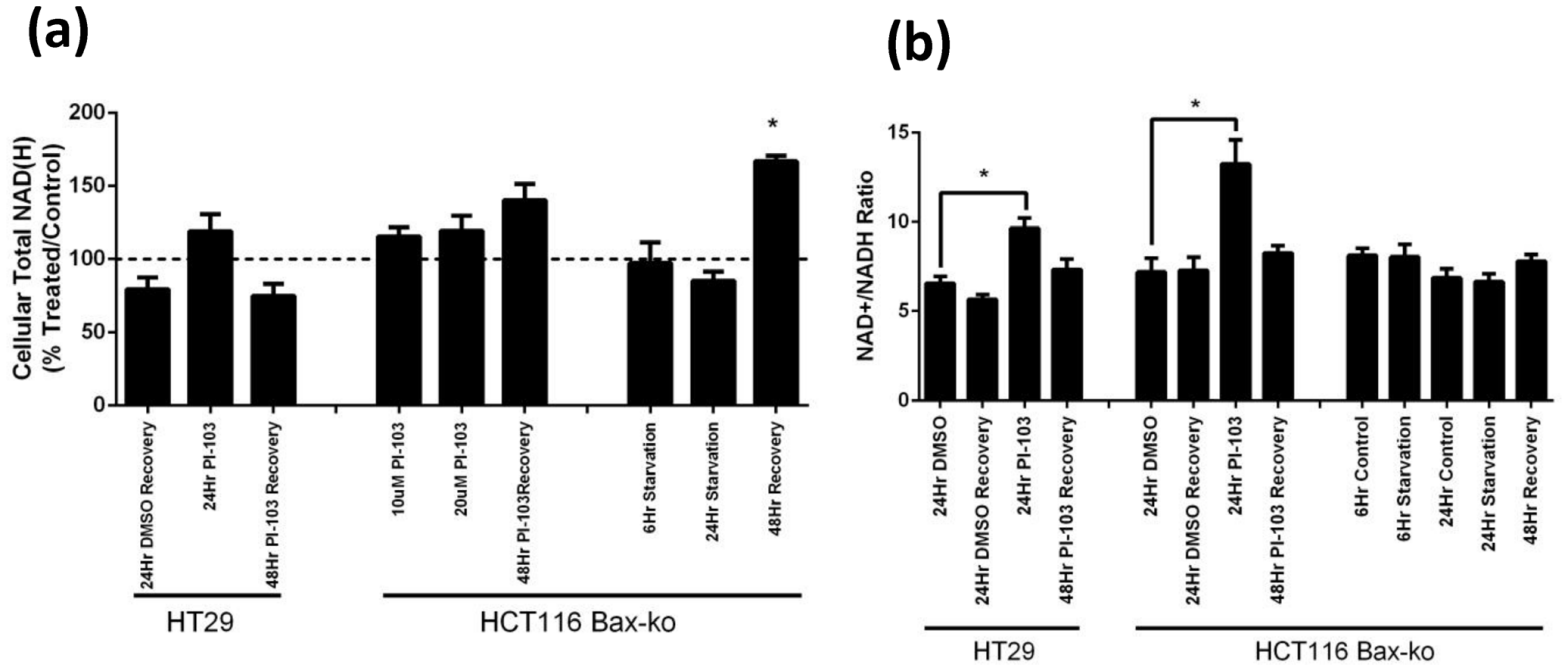
Levels of total NAD(H) pool and total NAD+/NADH ratios in PI-103- and HBSS-treated and its recovery in HT29 and HCT116 Bax-ko cells. (a) total cellular NAD(H) pool. Data are expressed as means of percentage ± s.e.m. (b) NAD+/NADH ratios in PI-103-treated or HBSS-treated HT29 and HCT116 Bax-ko cells and levels at 48 hours recovery from 24 hr of PI-103-treatment or starvation. Data are expressed as mean± s.e.m. Minimum *n* = 3 in each group. Statistically significant changes are indicated (*p≤0.05).

Intracellular NAD(H) levels were measured in cell extracts of each treatment group by ^1^H-MRS. No significant change in NAD(H) was observed in HT29 and HCT116 Bax-ko cell extracts after PI-103 treatment and 48 hr of recovery, or 6, or 24 hr starvation in HCT116 Bax-ko cells (Figure 7a). Significantly increased NAD(H) levels (167±4% of control; p<0.0001) were found in starved cells following 48 hr of recovery (Figure 7a).

Levels of NAD+ and NADH were further examined using a NAD+/NADH kit and NAD+/NADH ratios were evaluated in all treatment and recovery groups (Figure 7b). Increased NAD+/NADH ratios were found in HT29 (148±10% of control; p = 0.01) and HCT116 Bax-ko cells (185±14% of control; p = 0.02) following 24 hr of PI-103 treatment and they returned to control levels by 48 hr of recovery (Figure 7b). No significant change in NAD+/NADH ratio was found in HCT116 Bax-ko cells following 6 hr or 24 hr starvation or 48 hr recovery from 24 hr starvation (Figure 7b).

### LDH activity was reduced in 24 hr starved cells and unchanged in PI-103-treated cells

**Figure 8:**
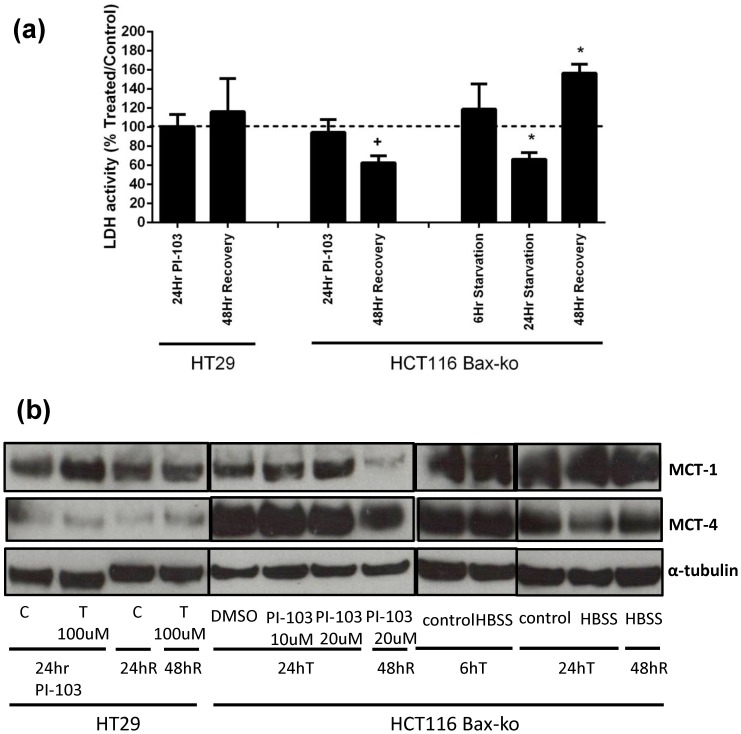
LDH activity and MCT-1 and MCT-4 expressions in PI-103- and HBSS-treated and its recovery in HT29 and HCT116 Bax-ko cells. (a) LDH activity ratios (treated/control) in PI-103-treated or HBSS-treated HT29 and HCT116 Bax-ko cells and levels at 48 hours recovery from 24 hr of PI-103-treatment or starvation. Minimum *n* = 3 in each group. Statistically significant changes are indicated (*p≤0.05). ^+^p = 0.06. (b) MCT-1 and MCT-4 expressions in PI-103-treated or HBSS-treated HT29 and HCT116 Bax-ko cells and levels at 48 hours recovery from 24 hr of PI-103-treatment or starvation.

Total cellular LDH activities were measured in all treatment and recovery groups. No significant change in total cellular LDH activity was found in HT29 and HCT116 Bax-ko cells following PI-103 treatment or after 48 hr of recovery in HT29 cells (Figure 8a). Reduced LDH activity was found in HCT116 Bax-ko cells following 24 hr of starvation (66±7% of control; p = 0.03) when compared with controls (24 hr full media-treated) and it increased above control levels following 48 hr of recovery (157±9% of control; p = 0.01) (Figure 8a). No change in LDH activity was seen following 6 hr starvation.

### Increased monocarboxylate transporter-1 (MCT-1) expression in PI-103-treated HT29 cells and lower MCT-4 expression with 24 hr starvation

MCT-1 and MCT-4 expressions in HT29 and HCT116 Bax-ko cells following PI-103 treatment and starvation and 48 hr recovery are shown in Figure 8b. An increase in MCT-1 expression was found in PI-103-treated HT29 cells and no change in MCT-4 was found in this group (Figure 8b). Reduced expressions of MCT-1 and MCT-4 were found in HCT116 Bax-ko cells recovered from PI-103 treatment. Expression of MCT-1 expression was found to return to control level in HT29 cells recovering from PI-103 treatment. Reduced MCT-4 expression was found in 24 hr-starved HCT116 Bax-ko cell and it returned to control level upon recovery (Figure 8b). No change in MCT-1 expression was found in any of the starvation groups. Our data indicate that a decrease in k_PL_ after PI103- treatment or starvation is unlikely to be a consequence of altered MCT expression.

## Discussion

We have induced autophagy in HT29 cells with PI-103 treatment and in HCT116 Bax-ko cells by two different methods, through serum and amino acid starvation using HBSS growth medium, or treatment with PI-103, confirmed by overexpression of LC3II in western blots and observation of increased numbers of autophagic vacuoles by electron microscopy. Significant reductions in adherent cell number were observed in all treatment groups compared with controls. Minimal levels of necrosis and apoptosis were seen by propidium iodide staining, annexin V and by western blots in all treatment groups compared with controls. Following 48 hr of recovery after 24 hr starvation or treatment with PI-103, LC3II returned to basal expression levels with increased cellular proliferation confirmed by the growth in the adherent cell fraction to greater than 24 hr vehicle-treated control values. Consistent with published reports [28], [29], our data also show that autophagy is a reversible process in response to stress and that cancer cells can recover from this process when the stress is removed.

DNP measurements showed significant reductions in k_PL_ in PI-103-treated or 24 hr starved cells but not after 6 hr starvation. Cells recovering following starvation exhibited a return of k_PL_ to a level above 24 hr vehicle-treated control rates. An increased k_PL_ was also observed in HCT116 Bax-ko cells recovered from PI-103-induced autophagy when compared with rates during treatment, but k_PL_ did not reach 24 hr vehicle-treated control rates. k_PL_ decreased further in HT29 cells after 48 hr of recovery from PI-103 treatment. Previous research has shown that k_PL_ is sensitive to a number of factors, including changes in LDH activity [19], the availability of the enzyme co-factor NAD(H) [14], changes in substrate concentration for example the endogenous pool size of lactate, modulation of monocarboxylate transporters [30] and NAD+/NADH ratios [31]. In our experiments the reduction in k_PL_ in PI-103 treated HT29 and HCT116 Bax-ko cells are associated with significant increase in NAD+/NADH ratio and decreases in intracellular lactate and lactate excretion. A recent report in breast cancer cells showed that the induction of autophagy and reduction in AKT/mTORC1 signaling pathway can be regulated by mitochondrial complex 1 through an increased NAD+/NADH ratio [32]. The observed increase in NAD+/NADH ratio following PI-103 treatment in our experiments is consistent with enhanced mitochondrial complex 1 activity leading to increased oxidation of NADH generated during glycolysis and the TCA cycle. The reduction in the Warburg effect during PI-103-induced autophagy could result in more pyruvate being diverted to the TCA cycle and a concomitant reduction in intracellular and excreted lactate. Enhanced TCA cycle activity during autophagy may also result in autophagic cells being less reliant on aerobic glycolysis, sustaining cellular homeostasis by utilizing autophagic degradation products to fuel cell metabolism [21]. This is consistent with the NAD+/NADH ratio returned to control levels following recovery from PI-103-induced autophagy in both HT29 and HCT116 Bax-ko cells. Our data are consistent with the known inverse relationship between lactate/pyruvate ratios and NAD+/NADH ratios [31], [33]. Previous reports showed that k_PL_ rates have a strong linear correlation with lactate/pyruvate ratios [34], [35] and therefore a reduction in k_PL_ would be consistent with a decreased lactate/pyruvate ratio and an increased NAD+/NADH ratio.

Reductions in k_PL_ were previously reported to be associated with reduced LDH activity due to PI3K/mTOR inhibition [19]. However, the AKT/mTOR pathway is inhibited in both cell lines in the present study following PI-103 treatment with no significant changes in LDH activity. The observed reduction in k_PL_ in PI-103-treated cells and associated increase in NAD+/NADH ratio is therefore likely to be a consequence of autophagy and processes associated with inhibition of the PI3K/mTOR pathway rather than solely resulting from pathway inhibition. Our findings further show that the previously reported reduction of k_PL_ [19] and those observed in the present study in response to PI3K/mTOR inhibition, can also occur in cells that will recover from treatment and are therefore potentially resistant to the treatment.

Following starvation induced autophagy, significantly decreased k_PL_, as well as reduced intracellular lactate and lactate excretion, were observed. In contrast to treatment with PI-103, there was no change in NAD+/NADH ratio, but reduced LDH activity in 24 hr starvation-induced autophagic cells was observed. This reduction in the Warburg effect during 24 hr starvation-induced autophagy could be attributed to LDH being sequestered and digested within the autophagic vacuoles [36], thus contributing to the reduction in LDH activity and to the reduced pyruvate to lactate exchange. The availability of nutrients in the media was very limited for starved cells making them more reliant on the digestion of intracellular organelles and proteins to sustain homeostasis. Hence, more cytosolic enzymes such as LDH may have degraded, leading to reduced LDH activity and Warburg effect. However, nutrients are freely available in the media to the PI-103-treated cells, which are thus able to take up and utilize the available nutrients, leading to a different metabolic response compared with starved cells. k_PL_, LDH activity and NAD+/NADH ratio remained unchanged in HCT116 Bax-ko cells after 6 hr of starvation, possibly reflecting an early stage in the formation of autophagic vacuoles and thus in the degradation of cellular proteins, organelles and cytoplasm.

In the cells recovered from starvation, the higher k_PL_ was associated with a recovery in lactate excretion and in intracellular lactate as well as with an increased concentration of cellular NAD(H) levels and of LDH activity, therefore enabling increased turnover of hyperpolarized substrates. This may indicate increased aerobic glycolysis to re-establish cellular homeostasis supporting recovery from starvation-induced autophagy. k_PL_ also increased in HCT116 Bax-ko cells recovering from PI-103-induced autophagy, although it did not reach the level of 24 hr vehicle-treated controls, which was consistent with a sustained reduction in lactate excretion at this time-point. These changes indicate that the cells may require a longer time for the reduced glycolytic effect to recover following PI-103-induced autophagy. The stored metabolites arising during the autophagy process may still be elevated and hence, an increase in metabolism is not yet required to replenish exhausted levels of substrates.

Reduction in Warburg effect was found in both HT29 and HCT116 Bax-ko cells undergoing PI-103- and starvation-induced autophagy, however, the mechanisms behind this metabolic response are different between drug-induced and starvation-induced autophagy. Overall, our data indicate that the reduction in the Warburg effect during starvation and PI-103-induced autophagy is reversible in cancer cells and enhanced even further in cells recovered from starvation-induced autophagy.

Our experiments demonstrated a significantly reduced pyruvate-to-lactate exchange in cells exhibiting treatment-induced autophagy, in the absence of significant apoptosis or necrosis. Day *et al.* showed reduced exchange of hyperpolarized [1-^13^C] label from pyruvate-to-lactate during apoptosis, attributed to reduced NAD(H), due to poly(ADP-ribose) polymerase (PARP) mediated depletion of the NAD(H) pool [14]. A reduced [1-^13^C] pyruvate to lactate exchange rate was also observed following inhibition of the MEK [18] and PI3K [19] pathways. The results of the present study indicated that reduced exchange of hyperpolarized [1-^13^C] label from pyruvate to lactate also occurs during drug-induced autophagy, suggesting an additional mechanism that must be considered when interpreting such data. Importantly, we found that cancer cells can recover from drug-induced autophagy leading to a potential mechanism for cellular resistance to therapies, resulting in a different treatment outcome compared with cells undergoing apoptosis. Our data indicated that cells recovering from autophagy are associated with an increase in k_PL_, which could provide a potential marker of resistance to treatment.

In conclusion, we have confirmed that a decrease in k_PL_ measured with hyperpolarized pyruvate and reduced steady state endogenous and excreted lactate measured with ^1^H MRS are a measureable result of the major metabolic changes accompanying starvation- and drug-induced autophagy in colorectal carcinoma cells. We have also shown that this can return towards and above control values with recovery. An increase in k_PL_ could potentially provide a marker for resistance to treatment. Future studies are required to confirm these findings *in vivo* and to examine the generality of our findings in different cancer cell lines. The measurements of the apparent rate constant k_PL_ of pyruvate to lactate exchange by DNP and ^13^C-MRS provides a valuable biomarker for the starvation- and drug-induced autophagic process in longitudinal studies, enabling real-time monitoring of the Warburg effect.
